# The Expanding Role of Traceability in Seafood: Tools and Key Initiatives

**DOI:** 10.1111/1750-3841.13743

**Published:** 2017-08-21

**Authors:** Sara G. Lewis, Mariah Boyle

**Affiliations:** ^1^ FishWise P.O. Box 233 Santa Cruz CA 95061 U.S.A

**Keywords:** seafood traceability, traceability initiatives, traceability tools

## Abstract

In the last decade, a range of drivers within the seafood sector have incentivized the application of traceability to issues beyond food safety and inventory management. Some of the issues motivating the expanded use of traceability within the global seafood sector include: increased media attention on the legal and social risks within some seafood supply chains, governmental traceability requirements, private‐sector sustainability commitments, and others. This article begins with an overview of these topics in the seafood industry, and why many nongovernment organizations (NGOs), companies, and government actors have turned to traceability as a tool to address them. We discuss how traceability connects to key requirements of environmental sustainability and social responsibility. Later, we review the range of traceability services, tools, software solutions, and the due diligence measures that are currently being leveraged within the seafood sector. The paper concludes with a discussion of several NGO‐ and industry‐led traceability initiatives that are examples of seafood traceability improvements.

## Introduction

Food safety regulation has been a major driver of the development and investment in traceability practices and technologies across most food sectors during the past few decades. Since the publication of the 2013 *Journal of Food Science* supplement on traceability (IFT 2013), examples of illegal harvesting of seafood and the mislabeling of seafood products have become more prominent in the media, and the risks they pose to consumers and businesses are more often being discussed. In addition, public sustainable‐sourcing commitments have been made by a growing number of restaurants, retailers, and suppliers of seafood around the world. In light of these developments, traceability is increasingly being applied within seafood supply chains to address a wider range of issues and concerns among consumers, seafood companies, government agencies, and the nonprofit sector about the legality and sustainability of seafood products. In developing this paper, we have conducted a review of peer‐reviewed sources, white papers, news reports, press releases, and websites relating to the topic of seafood traceability, particularly those that discuss how traceability connects to key requirements of environmental sustainability and social responsibility. We outline below several of the most prominent issues motivating the use of traceability within the global seafood sector.^1^

**Sustainability commitments** have become more common among seafood businesses in the past decade, and many companies are looking to traceability as a tool that will help them track progress towards their commitments and help to confirm that environmental goals have been met. Many environmental groups working in marine conservation are pointing to the importance of robust traceability mechanisms to identify socially‐ and environmentally‐responsible seafood. For instance, the Conservation Alliance for Seafood Solutions (2017), an alliance of North American marine conservation nonprofit organizations, has developed a “Common Vision for Sustainable Seafood” which states that, “understanding your products and where they come from enables you to assess the sustainability of your products, measure changes, and take action to improve supply over time.” In addition, Greenpeace's 2015 Carting Away the Oceans report (IX) scores retailers on purchasing policies, red list sales, participation in reform initiatives, and transparency, and states that seafood should be traceable from the water to the point of sale (Greenpeace 2015).

**Fishery improvement projects (FIPs)** have been developed as a means of transitioning unsustainable fisheries into sustainable ones. Leveraging the power of the private sector through multistakeholder collaboration, FIPs seek to address environmental and management challenges via the implementation of time‐bound improvement plans (Conservation Alliance for Seafood Solutions 2015). Because FIP products have the potential to meet the responsible procurement policies of some companies, there is now a need to incorporate traceability practices to ensure FIP products are correctly identified, and that the fishery can support improvement claims. In response to this need, a growing number of improvement projects are investigating how best to incorporate traceability goals into their work plans.

**Seafood fraud**—such as knowingly mislabeling species name—is not a new phenomenon, but was until relatively recently thought to occur so rarely that it would have little impact on consumers. In 1991, the U.S. Food and Drug Administration (FDA) established the Office of Seafood and began conducting more frequent inspections (Foulke 1993). Tests revealed that from 1988 to 1997 an estimated 37% of fish and 13% of other seafood were incorrectly labeled (Tennyson and others 1997). Since then, seafood production has continued to globalize and supply chains have become increasingly complex, providing further opportunities for fraud to occur. DNA tests conducted by nongovernment organizations (NGOs), journalists, and government bodies have shown that mislabeling and fraud occur globally, with instances identified in the U.S., South Africa, Europe, Australia, Brazil, and Hong Kong among others (Center for Food Safety 2007; von der Heyden and others 2009; Garcia‐Vazquez 2011; Espiñeira and Vieites 2012; Miller and others 2012; Warner and others 2016). The consequences of mislabeling can be serious as some species of fish that are used as substitutes can put consumers at risk of negative health impacts such as allergies; and intentional species substitution and mislabeling can be used to bring illegally caught fish to market (NMFS 2016; Warner and others 2016). DNA testing is a good way to confirm species claims for products, and can be used as a verification spot‐check within the framework of an end‐to‐end interoperable traceability system (FishWise 2016).

**Illegal, unreported, and unregulated (IUU) fishing** is a global problem that results in estimated fishing loss of $10 to 23.5 billion per year, representing 11 to 26 million tons of seafood (Agnew and others 2009). In the Pacific tuna fishery alone, recent estimates place the overall value loss to IUU fishing at approximately $616.11 million U.S. dollars, with approximately one‐fourth of that loss coming from reduced employment and access fees for the small nations of the Pacific (MRAG Asia Pacific 2016). Revenue losses from illegal and unreported harvest can affect some countries greatly (for example, 40% of West Africa's total catches may be illegal), and in some places illegal and undocumented fishing can be double the documented harvest numbers (Agnew and others 2009; Pramod and others 2014; Africa Progress Panel 2016). Illegal fishing can also lead to localized biodiversity loss and decreased food security, particularly when it pushes harvest levels beyond natural carrying capacities or uses unsustainable fishing methods (FAO 2016a). Further, IUU products can find their way into local and international markets where they may unfairly compete with legal products. In the United States, it has been estimated that as much as 20% to 32% by weight or $1.3 to 2.1 billion dollars of total value of wild caught seafood imports are from IUU sources (Pramod and others 2014). Implementing end‐to‐end interoperable electronic traceability is one way companies can improve access to information about the legality of the products they handle. Traceability systems can make it more difficult for illegal or undocumented products to enter supply chains if standardized data fields are requested and the accuracy of reported data is subsequently verified (EPLAT 2015; Naaum and Hanner 2016).

**Human rights abuses** such as forced labor and human trafficking have also received widespread attention in recent years—with reports linking seafood supply chains in 55 countries on 5 continents to forced labor (Mason and others 2015). Fishing and seafood processing have been tied to human rights issues such as unsafe working conditions, physical abuse, little to no pay for workers, and trafficking of fishers and children (EJF 2010; Surtees 2012; EJF 2013; ILO 2013; Stringer and Simmons 2013; USDOS 2016).Although these human rights abuses are at times occurring on vessels fishing illegally, many documented abuses occur within otherwise legal supply chains (UNODC 2011). The mainstream media has exposed abuses in aquaculture supply chains, aboard isolated fishing vessels at sea, and in many parts of seafood supply chains, including seafood processing (Hodal and others 2014; Mason and others 2015; Urbina 2015). First‐hand reports from survivors of trafficking and forced labor have attested to the problems that can result when unethical employers seek to offset high overhead and overexploited fisheries with inexpensive labor (Hodal and others 2014). Meanwhile, the logistical difficulties inherent in monitoring at‐sea working conditions within an increasingly globalized seafood industry allow trafficking and forced labor to persist (Marschke and Vandergeest 2016). On land, less visible links in seafood supply chains, such as local processing (for example, shrimp peeling sheds), can be overlooked as a result of weak regulations or weak enforcement (Mason and others 2015). Traceability systems can be used to collect and share information about the social/labor conditions that affect seafood workers in various supply chains (Bailey and others 2016).

## Governmental Traceability Requirements Are Expanding

In light of these concerns, nations are beginning to develop and implement regulations that make it harder for seafood products associated with illegal, fraudulent, or abusive practices to enter their markets; and they are increasingly mandating traceability measures to collect and verify information that they hope will improve the likelihood that illegal products will be detected. For instance, the 2 largest global markets for seafood imports—the European Union and the U.S.—have adopted regulations to combat IUU fishing and seafood fraud by implementing landmark traceability programs.

The EU has implemented regulations that only authorize imports from countries that ensure their fish and fishery products caught and processed outside the EU are in compliance with a food safety regulatory framework that is equivalent to that of the EU member states (EC 2016). The regulations request a Health Certificate and data availability in regards to the handling and food safety practices of all imported seafood. Since 2010, they also require catch certificates for every consignment imported to the EU. The catch certificates provide official assurances that the fish were harvested in accordance with applicable laws, international regulations, and management measures within the flag state (EC 2016). The certificate contains information about the product's catch vessel, transport vessel, scientific name, and FAO catch area, among others (EC 2016). The legislation behind the Catch Documentation Scheme also empowers the EU Commission or their designated representative to conduct audits to verify the effective implementation of flag state data verification arrangements.

In the U.S., the new Seafood Import Monitoring Program will apply beginning January 2018 to all at‐risk seafood entering U.S. commerce (NMFS 2016). The National Oceanic and Atmospheric Administration (NOAA) has identified a list of seafood species considered at‐risk for IUU fishing and seafood fraud (IUU Task Force 2015). The importers of record for those products will need to produce additional mandatory information pertaining to the harvest, landing, and chain of custody of the products prior to entry into U.S. commerce. Unlike the European system that still allows catch certificates to be submitted on paper, data collection for the U.S. Seafood Import Monitoring Program will occur via an electronic portal that allows information to be screened for completeness electronically. Information will be verified via random audits, at which time importers may be asked to produce additional traceability documentation for products (NMFS 2016).

Agreements promoting government‐to‐government information sharing are important signs of shifts towards traceability improvement in seafood. Japan, the 3rd largest consumer of seafood after the EU member states and the U.S., does not presently have any government‐mandated traceability requirements for all fish products—only labeling requirements within the Quality Labeling Standard for Perishable Foods (2000). Unprocessed products (including fish) need to be labeled with the product name, country of origin, wild/farmed designation, and fresh/frozen designation. Requirements for processed products, including fish (for example, fillets), are slightly more detailed and differ for domestically‐produced and imported products. In addition, Japan has signed joint statements against IUU fishing with the U.S. and the EU, committing to “systematically exchange information on IUU activities” with the latter (EC 2014; NOAA 2015). An agreement was also signed committing Japan to combat IUU fishing with Russia, and beginning in December 2014 Japanese ports began only accepting Russian fish accompanied by authorization certificates issued by the Russian Federal Fisheries Agency (Undercurrent News 2014).

These and other emerging national regulations appear to indicate a growing view among regulators that traceability can be an effective means to identify risk, improve data availability, reduce illegal imports, and hold flag nations more accountable for the actions of their fleets. Further, these regulatory requirements give seafood companies an incentive to begin implementing traceability protocols within their supply chains, and to establish data collection systems to manage the sharing of information about the identity, attributes, and sources of seafood products (information also known as key data elements [KDEs]).

This article describes some of the tools and services that exist for addressing various seafood sector traceability challenges, and provides an overview of some traceability initiatives. In response to the risks and opportunities described above, seafood businesses are increasingly turning to traceability as a tool to support the fulfillment of regulatory obligations, reduce liability through due diligence, protect brand integrity and company reputations, and reassure customers that their supply chains can be trusted. However, the traceability landscape can be difficult to navigate. There are many reasons why all members of the seafood industry do not already have end‐to‐end traceability via electronic interoperable systems—the best practice for seafood traceability. These include cultural, technological, and financial constraints such as remote geographies, technology costs and functional limitations, language barriers, and regulatory differences. For a more thorough discussion of the challenges to end‐to‐end interoperable seafood supply chains, refer to the article—Current Barriers to Large‐scale Interoperability of Traceability Technology in the Seafood Sector—by Hardt and others (2017) on page 3 of this Supplement.

## Summary of Traceability Services and Tools

As traceability is applied to a growing number of issues, the list of services and programs offered by organizations also continues to grow. However, there are often differences in the traceability needs of companies depending on their size, location, the types of products they sell, and their position within supply chains. Companies selling seafood to retail customers or consumers at the end of the supply chain have different data collection needs and challenges than those working near the point of harvest—largely due to the type and volume of information required to do business, and their physical environment. For instance, harvesters may struggle with collecting and storing reliable data about their catch while in remote locations or rough conditions, so they may look for traceability tools that will stand up to the elements while at sea or in transit. Aquaculture facilities may also be asked by customers to be accountable for monitoring and sharing information about all aquaculture inputs (for example, feed sources and quality) and other data down the supply chain. On the other hand, the greatest challenge for those businesses selling to consumers may be tracking information about a very large volume and array of products. Meanwhile, those in the middle of the supply chain (for example, processors) often require traceability systems for tracking the transformations they make to the seafood products (that is, changes to the weight, composition, or packaging of products) and other KDEs requested by their customers. Traceability tools and information can also support different purposes or objectives within each company in a supply chain—with those working on the upstream using traceability to support product quality and integrity claims, and those on the consumer side using it to educate their customers about their products or ensure compliance with due‐diligence policies.

This section provides a high‐level overview of data collection and information management tools that can be used to improve seafood traceability. These tools can be used in combination and customized to serve the specific business needs of companies.

## Harvester Tools

### Vessel monitoring technologies

Remote monitoring of fishing vessel activity is one way some private companies and governments have addressed the need to identify the location, dates of fishing and transshipment, and identity of fishing vessels. Vessel monitoring systems (VMS) typically use on‐vessel automatic identification system (AIS) transceiver units. The units transmit information about the location and movement of commercial vessels via electronic information exchange with other ships, base stations, or satellites to governmental or private recipients. AIS transceivers are required under the International Maritime Organization's (IMO) International Convention for the Safety of Life at Sea (SOLAS) for all passenger ships and for commercial vessels over 300 gross tons, although fishing vessels are currently exempt from this requirement (SOLAS Annex 17; IMO 2017). AIS transceivers are required, however, by many Regional Fisheries Management Organizations (RFMOs) and the fisheries management agencies of many countries (FAO 2017). For vessels that will remain close to shore, mobile phone technology can also be used to transmit information to relevant government bodies (U.S. Coast Guard 2017).

Information collected via VMS is highly confidential and tightly regulated, but can be used as a surveillance tool to deter fishing violations as part of a harvest management system (for example, NOAA 2016). Vessel tracking tools like VMS also have a broad range of other potential uses for traceability apart from generating fishing vessel data. For instance, VMS can be used to verify other sources of information about fishing activity. By comparing vessel tracking information to captain's logs, catch forms, or other documents, users can bolster confidence in the accuracy of reported dates of harvest, catch locations, and harvest vessel identity (Global Fishing Watch 2016; Pew Charitable Trusts 2017).

Although many governments employ VMS systems as part of fisheries management plans, many only require them for vessels above a minimum tonnage or capacity (FAO 2017). This is despite the fact that the technology is now also compatible with the scale and geographies of many small‐scale artisanal fisheries. With the development of small, lightweight, and durable transceivers that can operate with only solar power, or use of cell‐phone technology, companies can find vessel monitoring solutions at almost any scale. Some producers are even choosing to share low‐resolution vessel tracking data with their customers or the public to help market their products and drive confidence in various claims about quality, sustainability, and legality (Gulf Wild 2017; Thisfish 2017).

### Electronic logbooks

Often used along with vessel tracking tools, electronic logbooks can help reduce misreporting at the point of harvest and make data available in near real‐time to supply chains. In the most basic form, e‐logbooks consist of a phone, tablet, or computer application that records and securely submits information about catch and fishing effort via electronic files (CADFO 2016). These systems can collect and transmit some data automatically at set time intervals or in near real‐time. Given information about the species being targeted, the location of the vessel, and the vessel's speed, the e‐logbooks can help develop an estimate of fishing effort by location. Furthermore, some e‐logbooks can be synced with waterproof electronic transmitters attached to the fishing gear to record gear deployment and fishing depth. Other on‐board technologies that sync with e‐logbooks can monitor temperatures inside storage areas to ensure product is kept cold enough to prevent food safety concerns and maintain quality.

### Electronic monitoring

Fishing observers have been deployed in many parts of the world to identify, quantify, and record the catches of fishing vessels (APO 2017). Observers play an important role in ensuring that vessels comply with fishing regulations, and that fishing impacts (such as incidental catch) are recorded and handled properly. However, employment as a professional observer can be difficult and dangerous, and observer programs can be expensive to operate. In an attempt to expand the number of vessels that are observed with independent review, catch cameras have been installed aboard some fishing vessels to record catch electronically (NOAA 2017). When the nets are pulled in, cameras record video of the catch, including its composition, any processing on board, the release of animals, and storage. In their most basic form, these cameras simply record the catch and crew, and once the vessel returns to shore a trained observer or member of a company's staff watches the tape and records the catch. More recently, however, “catch accounting systems” that can automatically count and identify the catch have been developed for select fisheries.

## Traceability Software Solutions

Best practice for seafood traceability is electronic data transmission from point of harvest to consumer via interoperable software (Badia‐Melisa and others 2015; Bhatt and others 2016). Increasingly, companies are eschewing paper documents, and are instead using computer hardware and software to help them capture and share data as seafood moves throughout complex and globalized supply chains. Hardware solutions (such as bar code scanners, radiofrequency identification tags, and so forth) are often employed to collect traceability or product quality data which has led to a growing market for electronic traceability software (Badia‐Melisa and others 2015).

Many traceability technology providers are relatively new to seafood compared to other commodities such as produce, where traceability technology and 3rd‐party vendors are better established (see Bhatt and others 2017, on page 22 of this Supplement). However, the last decade has seen tremendous growth in the number of companies providing traceability software for seafood data capture, sharing, and tracking. These systems can operate within a single company or be linked to track products throughout a supply chain (harvest to point of sale). Traceability software typically works by storing and cross referencing KDEs about each traceable unit (whether a batch, shipment, or item) and generating unique identifiers that can travel with the product throughout its chain of custody. Many of these systems can seamlessly share information with customers, and can be implemented to meet the goal of end‐to‐end electronic interoperable traceability. Companies look to implement 3rd‐party software solutions for traceability when the return on investment (ROI) is higher, or implementation is easier, than incorporating KDEs and traceability information into their internal data systems (often enterprise resource planning [ERP] systems). These software solutions are often cloud‐based and can incorporate key sustainability or other corporate social responsibility components with encryption protocols for added data security (for more information on traceability software, see Hardt and others 2017, on page 3 of this Supplement). For seafood, those additional details could include electronic‐logbook data, farm or vessel identity and licensing information, food safety and storage data, aquaculture feed sources and ingredients, information on environmental, social, or traceability certifications for products, and many others.

## Due Diligence Measures

### Risk identification

If supply chains are data‐rich and standardized KDEs are shared between companies, the data about product sources and attributes can be used to assess and quantify risk. Risk can be identified by comparing KDEs against publicly‐available reports assessing levels of IUU fishing, labor abuses, or other factors (Yasuda and Bowen 2006; Fair Labor Association 2017). An advanced level of risk identification could entail programming a set of algorithms into data systems to automatically scan information for anomalies or flag data for review. For example, warnings can be triggered when product attributes are outside of standard levels (for example, pre‐ and post‐processing weights or volumes; Yasuda and Bowen 2006). Systems can also be programed to verify supply chain data against public databases, allowing the user to receive relevant notifications. For instance, if certifications are expired or invalid or if a vessel name appears on an IUU blacklist the program will send businesses alerts that allow them to investigate further.

### DNA testing

DNA testing has been used as a tool for authenticating seafood species identification and exposing seafood mislabeling and fraud (Warner and others 2016). Service providers take samples at any point from harvest to point of sale, and compare the genetic profile of the samples to reference profiles. For high‐value species such as bluefin tuna, some companies have used DNA fingerprinting to trace individual fish through supply chains. DNA testing has also been used to confirm if a product was genetically modified, raised with aquaculture or is from wild capture production, and to determine if it was assigned the correct market name (Naaum and Hanner 2016). In the past, it was only possible to test unprocessed fish, but it is now possible to test samples that have been frozen or processed (for example, battered or cooked; Naaum and Hanner 2016).

### Verification

Although it is important that traceability systems comply with data collection best practices, it is also critical that those systems and data are tested for accuracy and robustness (EPLAT 2015). There are numerous ways to do this including auditing supply chain companies against a set of practices or a standard, or spot‐checking product attributes (FishWise 2016). Company audits—often including a site visit—are useful because they provide insight into a company's operations. However, audits provide only a snapshot in time of a company's processes and should be used in combination with other verification measures. Other measures might include a traceability audit for a specific product (also known as traceback or mock recall). Tracebacks can reveal areas where traceability improvements are needed. For instance, some products may be found not to be traceable past a certain link in the supply chain, although other tracebacks could simply reveal minor inconsistencies in the data. Risk assessments are an excellent way to prioritize products for verification. Both tracebacks and audits are resource intensive, so it can be helpful to combine these with less time‐intensive verification spot‐checks. Spot‐checks can include the verification of one KDE—for example, confirming that a company holds a chain‐of‐custody certification via the certifier's website, searching online for a fishing vessel's history, or confirming that a fishing vessel name does not appear on a blacklist (EPLAT 2015). Other spot‐check measures include the DNA testing discussed previously, and many others that are often dependent on the supply chain (Naaum and Hanner 2016).

## Consumer Facing Traceability

Traceability information is increasingly leveraged for marketing and promotion purposes. Consumers are becoming more attracted to the story behind their seafood than the sustainability or nutritional aspects of the products, and some companies are tapping into opportunities for point‐of‐sale story telling about a product's source (FoF 2016a). When companies implement end‐to‐end interoperable electronic traceability tools they can choose to share more information with their customers and the public via social media, company websites, or in stores (Sterling and others 2015). Private companies and 3rd‐party traceability software solutions can build web‐based interfaces that are integrated with supply chain traceability tools to provide consumers with transparency about the source of their seafood and even biographies of the fishers that harvested it. By linking KDEs with unique identifiers, these systems can identify products by shipment, batch/lot, or even individual fish. Customers can view the traceability information on package or through applications on their phone, by scanning a QR code, or by entering a code on the company's website (FoF 2016b,c).

## Traceability Initiatives

Although the range of tools and services for seafood traceability is expanding, end‐to‐end interoperable electronic traceability has not yet been widely implemented. At present, many companies are still only sharing and receiving information with their direct trading partners via paper documentation or basic electronic recordkeeping systems. More detail on the slow adoption of interoperable systems is provided in the article—Current Barriers to Large‐scale Interoperability of Traceability Technology in the Seafood Sector—by Hardt and others (2017) on page 3 of this supplement. However, industry‐led initiatives, precompetitive collaborations, public–private partnerships, and NGO engagement with the private sector are advancing traceability improvements. We highlight some positive examples from the seafood industry here.

### Traceability work within the seafood industry

#### Industry trade groups

Several years ago, the National Fisheries Institute (NFI) worked with GS1 . United States to create a U.S. Seafood Traceability Implementation Guide (NFI and GS1 2011). Subsequently, in 2014, the NFI Traceability Working Group created a draft standardized list of data to be collected and shared within supply chains, or KDEs for identifying seafood sources. The KDE project was intended to define minimum requirements for information sharing and build industry consensus for standardized KDEs (FishWise 2015).

The Food Marketing Institute (FMI) is an organization representing food retailers and wholesalers. FMI's Sustainable Seafood Committee has developed resources and guidelines that will support stakeholder collaboration on seafood sustainability. In 2012, FMI released a Sustainable Seafood Toolkit to assist food retailers with the integration and implementation of seafood sustainability procurement policies and procedures (FMI 2012). The Toolkit provided examples and identified components that may be taken into consideration when developing policies. The importance of traceability is cited in almost all examples in the Toolkit, which were generated from meetings and discussions with members of the Sustainable Seafood Strategy Committee, interviews with industry leaders, and a review of industry best practices.

#### Seafood retailers

Retailers have also been recognizing the importance of seafood traceability and using it to track performance against their sustainable seafood commitments. For instance, Target (Minneapolis, Minn., U.S.A.) and the Midwest grocery chain Hy‐Vee (West Des Moines, La., U.S.A.) each made public commitments to improve the sustainability and traceability of their fresh and frozen seafood assortment by the end of 2015. Both companies reached milestones in their data collection improvements, risk assessment of products, and communication of traceability expectations with their vendors. Further, in 2016 Albertsons Companies (Boise, Id., U.S.A.), the 2nd largest retail grocer in North America, made a public traceability commitment that applies to all seafood categories from shelf‐stable tuna to those in the fresh/frozen case. Other retailers incorporating traceability into their seafood procurement are Ahold Delhaize Group's (The Netherlands) U.S. Hannaford and Food Lion stores, who say in their 2015 sustainability report that their private‐brand seafood products are traceable to the fishery or farm of origin (DelHaize Group 2015), and Wegmans (Rochester, N.Y., U.S.A.) markets, which have adopted interoperable traceability technology for their seafood (Wegmans Food Markets 2015).

#### Private seafood producer and supplier companies

Private companies making public commitments to improve the traceability of seafood include Thai Union Group (Samutsakorn, Thailand), which has publicly committed to “full traceability for all seafood (they) purchase” by the year 2020 (Thai Union 2016). Thai Union's 2015 sustainability report says the company is working on traceability in order to “reduce the risk of IUU fishing and … ensure every vessel complies with our stringent labor regulations” (Thai Union 2015). Seafood producers large and small have also begun to explore ways to communicate traceability information all the way to consumers. For instance, in 2009 Kwik'Pak Fisheries (Anchorage, Alaska) became one of the 1st wild salmon producers to adopt electronic, interoperable traceability software that enables customers to learn about the origin of the salmon they purchase (Kwik'Pak 2016). Since 2015, 2 of the largest national brand tuna companies in North America—Bumble Bee (San Diego, Calif., U.S.A.) and Chicken of the Sea (Mt. Olive, N.J., U.S.A.)—have created traceability features that allow consumers to trace their canned tuna through individual codes printed on each can (Bumble Bee Seafoods 2016; Chicken of the Sea 2016).

Seafood processor and distributor NorPac Fisheries Export, has under the leadership of founder Tom Kraft, developed and implemented a traceability software solution to trace product from point of harvest to the retailer and made that proprietary technology available for use throughout seafood supply chains via Insight Solutions. In 2014 NorPac, Insight Solutions, and the Nature Conservancy launched an electronic traceability pilot program with one of Indonesia's largest tuna processing plants. The program has demonstrated how electronic traceability tools and hardware can result in efficiency gains for private‐sector businesses, and that information can be collected and shared in a way that supports sustainable fisheries management (Undercurrent News 2015). Gulf Wild, a nonprofit organization supporting U.S. fishermen operating in the Gulf of Mexico, has developed a trademarked seafood brand that integrates gill tags with traceable QR codes. The tags stay attached as the product moves throughout the supply chain—allowing customers to access identifying information about the vessel, captain, and on‐board observers. Sharing traceability information end‐to‐end empowers consumers to uncover information such as the species, catch location, catch method, and processing location of their product, allowing them to make more‐informed purchases.

Precompetitive Collaborations. Companies and industry associations are also recognizing that greater leverage and collective impact can be obtained by working together. Precompetitive collaboration can lead to improvements that a single company would struggle to achieve without the buy‐in or financial support of other leading companies. One example is Sea Pact, formed by 6 North American Seafood Companies working towards improvement of social, economic, and environmental responsibility throughout global seafood supply chains. Sea Pact contributes financially to projects that meet its mission, such as one that is working to build a traceability system within a Brazilian lobster FIP (Sea Pact 2016).

The Seafood Task Force, formerly the Shrimp Sustainable Supply Chain Task Force, is an industry‐led precompetitive group with full supply chain participation (SSSCTF 2016). Focused on action and results, the Task Force was formed to address the risks of forced labor, human trafficking, and IUU Fishing in Thailand's seafood supply chain. According to Humanity United, the Task Force is the most influential and diverse coalition of stakeholders operating in Thailand on this issue (Stride and Murphy 2016). The group consists of seafood retailers, suppliers, major Thai processors, and feed companies. NGOs participate on an External Stakeholder Advisory Group, providing expert technical expertise in the areas of social responsibility, environmental sustainability, and traceability. The Task Force's work to date includes supply chain analysis, identifying the vessels harvesting the inputs for fish feed, development of audit protocols, and work with the Thai government to develop port control measures and documents. One of its main objectives is to implement traceability systems with international verification from vessel to feed mill, and have this system become an independent, internationally‐recognized benchmark supply chain model within the industry.

## Nonprofit Initiatives

Non‐profits are playing an important role in advancing the conversation and understanding of seafood traceability through development of informational reports, guidelines, and collaborative efforts. Although there are numerous groups working on some aspects of traceability, 4 have been working together during the past few years to help shape traceability innovation via the Oceans and Seafood Markets Initiative Seafood Traceability Collaboration. These NGOs are working closely together to promote seafood traceability by engaging companies and producing educational tools and resources for businesses.

FishWise (authors of this paper) is a nonprofit sustainable seafood consultancy working with North American retailers and mid‐supply chain companies to improve sustainability, traceability, and human rights in their seafood supply chains. Uniquely positioned between the seafood industry and marine conservation organizations, FishWise plays a critical role in connecting and convening a network of companies, nonprofits, and policymakers involved in key traceability projects and precompetitive collaborations. FishWise's contributions to the space include integrating traceability and human rights into seafood sustainability discussions, equipping the Conservation Alliance for Seafood Solutions with tools and resources to better promote traceability, working with U.S. companies to implement ambitious but achievable traceability and social commitments, and creating tools and resources to improve traceability implementation by the industry and advance stakeholders’ shared understanding of traceability.

Future of Fish (FoF) is a design‐process expert–driving collaboration, prototyping, and storytelling in seafood. Through its pilot project work FoF has become an expert in understanding the granular details of traceability technology deployment in the field and the technical challenges that can occur. It has also built unique relationships with technology vendors to allow them to coordinate and voice support for a growing demand within the seafood sector. FoF's specific achievements include developing initiatives that get traceability technology companies together to discuss challenges in a precompetitive environment. It has also worked to refine the business case for traceability at all levels of the supply chain, and created compelling education tools for the NGO sector. In 2014, FoF released Getting There From Here, a report comparing seafood traceability technology providers, and in 2016 produced a Traceability 101 Toolkit for NGOs and businesses wanting to deepen their knowledge of traceability (FoF 2014, 2016b). FoF's Technology for Transparency Pod is now working with members of the seafood industry and technology providers to understand where opportunities lie to improve data movement and retention in seafood supply chains, improve verification, and “keep story attached to fish, truth (and) transparency” (FoF 2016c).

The Inst. of Food Technologists’ (IFT) Global Food Traceability Center (GFTC) is an internationally renowned institute for food traceability. Working across food sectors allows the GFTC to bring unparalleled insights about technology application and transparency adoption in other food systems and supply chains to work with seafood traceability. The GFTC is comprised of experts in both traceability “rollout,” as well as the strategy and execution of building industry‐wide training and standards dissemination. GFTC's website includes numerous traceability resources relevant to seafood businesses, such as the “Impacts of Traceability on Business Performance,” “Seafood Consumer Preference Tool,” “Seafood Traceability Financial Tool,” and an issues brief on “Interoperable Seafood Traceability Technology Architecture,” among others (GFTC 2015a, b; Sterling and others 2015; Bhatt and others 2016; GFTC 2016).

The World Wildlife Fund (WWF) is a global conservation NGO that, through its policy level leadership and partnerships across sectors, creates connections between the business and nonprofit worlds to align and work toward conservation goals together. WWF is currently working in multiple countries to improve the sustainability of fisheries. They created a “Traceability Principles” manifesto to align the NGO world around traceability, and piloted the development of supply chain risk assessment and traceability benchmarking tools (WWF 2015). In 2013, the WWF convened an Expert Panel on Legal and Traceable Seafood Products, a multidisciplinary group of experts on traceability, IUU fishing, and sustainable fishing. In March 2015, they published a report outlining recommendations for a global traceability framework (EPLAT 2015). Subsequently, in April 2015 WWF released a set of guidelines with Traceability Principles for Wild‐Caught Fish Products (WWF 2015). WWF and the IFT GFTC are spearheading a project to create a traceability architecture to allow for improved electronic and interoperable seafood traceability. They colead the Global Dialogues on Traceability, helping to ensure that a system is created which can be applied to seafood from all regions and supply chains.

## Public Sector Initiatives and Public–Private Partnerships

The U.S. State Department, U.S. Agency for International Development (USAID), and The Association of Southeast Asian Nations (ASEAN) have established a 5‐year (2013 to 2018) Oceans and Fisheries Partnership. One goal of this partnership is to design and implement a catch documentation and traceability (CDT) system in seafood supply chains that will align with the FAO's best practices, combat IUU fishing, and take an ecosystem approach to fisheries management. The partnership aims to design a standardized, electronic, interoperable, transparent CDT system for priority species in the region (USAID 2016).

The FAO has also initiated work towards the creation of a “Global Record of Fishing Vessels, Refrigerated Transport Vessels and Supply Vessels.” The Global Record is intended to be a tool for improving global transparency and traceability in the fisheries sector that will complement existing efforts, such as the United Nations’ Port State Measures Agreement (PSMA) and the FAO's Voluntary Guidelines for Flag State Performance (FAO 2016b). Fishing vessel registration and the maintenance of a comprehensive record of fishing vessels are fundamental for both effective fisheries management and effective collaboration among governments and within supply chains. The FAO's Global Record Working Group seeks to address the lack of transparency and traceability in the fisheries sector that enables perpetrators of IUU fishing to easily sell their products in legitimate markets. The goal of the Global Record is to help deter and eliminate IUU fishing by making it more difficult and expensive for vessels and companies acting illegally to do business (FAO 2016b).

## Cross‐Sector Collaborations

Global Fishing Watch, a partnership between SkyTruth, Oceana, and Google, is a new, interactive technology platform that enables anyone with an internet connection to view global commercial fishing activity in near real‐time. It seeks to empower stakeholders by providing transparency, which in turn will help drive the research, advocacy, policy‐making, monitoring, and enforcement needed for the effective management of fisheries and oceans. Global Fishing Watch can also serve as a simple and inexpensive way for fishermen to demonstrate they are fishing responsibly (Global Fishing Watch 2016).

The Pew Charitable Trusts and the Satellite Applications Catapult have developed “Eyes on the Seas,” a technology platform that seeks to improve ocean sustainability through actionable insight of global fishing activities, legal, and illegal. The platform uses multiple data sources, including satellite data, fishing vessel databases, and oceanographic data, to survey vessel activity at sea and alert fisheries analysts to suspicious activity. The software then compiles information from these many sources into data‐rich images in near real time (Pew Charitable Trusts 2015a). Fisheries monitoring and enforcement agencies can use the system to identify illegal fishing activity, or independently verify reported “at‐sea” activities. The project also aims to provide information to governments and industry players who are interested in understanding in more detail fishing activities in their waters or supply chains (Pew Charitable Trusts 2015b).

## Discussion and Conclusion

Each seafood company is unique, and as with food safety management there is no one‐size‐fits‐all approach for traceability. Instead, each company can approach traceability improvement by first becoming informed about traceability best practices, what types of traceability services are available, and what they can learn from other companies or initiatives (FishWise 2016). Whether a company is engaged in the harvest of seafood or its final sale there are a number of traceability tools and services that can be leveraged to support food safety, track progress against sustainability commitments, and improve data sharing among supply chains (FoF 2015). By better understanding and managing their supply chains and tracking specific products back to their source, companies can reduce or eliminate risks while maximizing inventory control (GFTC 2015a). Investing in solutions now will help companies to protect brand value, build consumer trust, and directly address environmental and social issues (GFTC 2015b).

However, the ROI companies get from traceability investment will vary from one company and supply chain to the next (Sterling and others 2015). In order to begin to improve supply chain traceability there are a few key steps companies can take. To begin with, any company wishing to improve the traceability of their products needs to clearly communicate their traceability expectations to their suppliers; and in most cases, it also helps to ask suppliers to share their current traceability practices (FishWise 2016). Vendor communications can go a long way to reveal where traceability risks and opportunities lie. Companies can work together to identify ways to improve their internal data systems or identify 3rd‐party software, and existing ROI tools can help identify the most valuable improvements (GFTC 2015b). Due to the rapid development in the seafood traceability sector, some of the traceability tools and services described above have become more economical, and businesses can see returns on their investments (GFTC 2015b). Finally, companies can conduct risk assessments and prioritize the highest‐risk products for additional review, via audit, spot check, or traceback.

Ensuring that seafood supply chains are fully traceable and that product is legal and accurately labeled is a significant undertaking, but it is a challenge that must be met head‐on if companies are to achieve their sustainability goals (EPLAT 2015; Conservation Alliance for Seafood Solutions 2017). This article has provided an overview of some of the tools and resources available to members of seafood supply chains—from traceability software solutions and other technologies that help companies collect and share information, to industry and NGO initiatives that can provide guidance or voices of experience to businesses looking to improve their supply‐chain traceability. As governments and international media focus more attention on eliminating IUU fishing and ensuring that human rights are protected, it will be important that seafood companies are also proactive on this topic. Improving the traceability of seafood products globally will require shared effort by governments, private businesses, and NGOs (EPLAT 2015; Sterling and others 2015).

## Disclosures

FishWise is funded by several foundations to improve traceability and combat illegal fishing and human rights abuses within seafood supply chains. FishWise also works with industry as a nonprofit consultant to help them set and implement responsible seafood policies and commitments.

## Footnotes

1 Although many of the examples in this paper focus on wild‐capture fisheries, the same drivers can often be observed within aquaculture supply chains.
